# QoE collaborative evaluation method based on fuzzy clustering heuristic algorithm

**DOI:** 10.1186/s40064-016-2459-z

**Published:** 2016-07-07

**Authors:** Ying Bao, Weimin Lei, Wei Zhang, Yuzhuo Zhan

**Affiliations:** Institute of Communication and Information System, School of Computer Science and Engineering, Northeastern University, Shenyang, 110819 China

**Keywords:** Quality of experience (QoE), Quality of service (QoS), Multipath transmission, End-to-end QoE evaluation system

## Abstract

At present, to realize or improve the quality of experience (QoE) is a major goal for network media transmission service, and QoE evaluation is the basis for adjusting the transmission control mechanism. Therefore, a kind of QoE collaborative evaluation method based on fuzzy clustering heuristic algorithm is proposed in this paper, which is concentrated on service score calculation at the server side. The server side collects network transmission quality of service (QoS) parameter, node location data, and user expectation value from client feedback information. Then it manages the historical data in database through the “big data” process mode, and predicts user score according to heuristic rules. On this basis, it completes fuzzy clustering analysis, and generates service QoE score and management message, which will be finally fed back to clients. Besides, this paper mainly discussed service evaluation generative rules, heuristic evaluation rules and fuzzy clustering analysis methods, and presents service-based QoE evaluation processes. The simulation experiments have verified the effectiveness of QoE collaborative evaluation method based on fuzzy clustering heuristic rules.

## Background

As the subjective evaluation by users on network transmission service, quality of experience (QoE) (ITU-T 2007) is widely used as the basis for media payload distribution and service transmission control, while quality of service (QoS) (3GPP 2004, 2007) is more utilized as an objective parameter for the QoE research, which refers to the network transmission capacity (e.g., delay, jitter, packet loss rate, bandwidth and other parameters). Currently, the methods of QoE evaluation for network transmission are mainly focused on end-to-end QoE evaluation system, with which parameters including QoS parameters and users’ subjective information are directly collected from end devices during the transfer period. The design model is mainly based on linear rules (Volk et al. 2010; Sterle et al. 2011; Yu et al. 2011). This kind of method realizes the modeling based on fixed parameters and helps obtain the score of QoE through linear rules programming. From a practical point of view, there is a certain difference between the actual user experience and the result of this QoE evaluation model. Besides, the evaluation model will not perform well when the expansion of parameters has been affected by media applications. Therefore, a reasonable and compatible QoE evaluation method, with which the quality of user experience could be accurately reflected, is needed to meet the requirements for the evaluations on user experience and various applications.

In this paper, a method of QoE collaborative evaluation (QoE co-evaluation) based on fuzzy clustering heuristic algorithm is proposed, which adopts a centralized-server management mode with the server as the core entity. In execution period, clients provide service QoE evaluation parameters to the server and cooperate with the server to complete the evaluation of QoE at the server side. At the same time, on the basis of the theory of fuzzy mathematics, artificial intelligence and knowledge discovery, a method of QoE collaborative evaluation for multipath transport based on fuzzy clustering heuristic algorithm could be further realized, which could be utilized for path selection, service score and payload distribution management of media transmission.

## Related work

### Analysis of end-to-end QoE evaluation method

The QoE guarantee system includes a whole end-to-end system, covering the user, terminal equipment, core network, access network, service infrastructure, etc. In addition to the QoS factors for end-to-end network, QoE is also affected by user subjective factor, terminal capacity, application properties, physical environment and other factors. The existing QoE evaluation method takes on various types from several aspects, which are mainly divided into three categories, namely subjective evaluation method, objective evaluation method, and the combination of subjective and objective method.

#### Subjective evaluation method

Subjective evaluation method is utilized to maintain the direct service evaluation information of end users [e.g., the one-click scoring method presented in Literature (Chen et al. 2009)]. The commonly used method of subjective evaluation is MOS (Mean Opinion Score) method proposed by IETF. The advantage of subjective evaluation is that user’s evaluation score could be directly and accurately collected. Nevertheless, it costs much and has a high requirement for the objective environment. Thus, it has not been widely used.

#### Objective evaluation method

Objective evaluation method is realized through the comparison between the output sequence and the original sequence of services. The existing research methods are mostly based on QoS parameters mapping, which will provide a relevant formula for QoS and QoE. Literature (Garcia et al. 2009) gives a service QoE evaluation model at the client side based on QoS parameters (such as delay, jitter, and packet loss rate), as shown in Formula (1).

1 $${\text{QoE}}_{\text{n}} = \frac{1}{{\left( {{\text{Delay}} + {\text{k}}_{1} {\text{Jitter}}} \right){\text{e}}^{\text{PacketLoss}} }}$$

Here, *Delay* and *Jitter* respectively represent the delay and jitter of k_1_nodes. *PacketLoss* denotes packet loss rate.

2 $${\text{QoE}}_{\text{u}} = \frac{{{ \log }_{{10\left( {{\text{v}}_{\text{q}} } \right)}} }}{{{\text{t}}_{\text{zap}} + {\text{K}}_{1} {\text{t}}_{\text{sync}} }}$$

wherein, *v*_*q*_ represents video quality, *t*_*zap*_ channel switching time. K_1_ means the degree of importance between *t*_*sync*_ and *t*_*zap*_. *t*_*sync*_ represents the synchronous error between video and audio. The different values of *QoE*_*n*_ and *QoE*_*u*_ are obtained by establishing the relationship between QoS parameters and QoE, so as to complete the QoE policy decision model eventually.

In Literature (Kim 2010), a QoE evaluation model for IPTV service is established. QoE related parameters and service model are achieved from the relationship between QoS and QoE, and service evaluation is executed at the client side. Nonetheless, this model only suits for the video service of IPTV. Literature (Sterle et al. 2011) presents a paired comparison method of service QoE quantitative evaluation, which records comparative result and get the QoE quantitative value with Bradley-Terry-Luce (BTL) models.

Objective evaluation method has many advantages such as high accuracy. However, it overemphasizes the importance of objective factors in the execution period, hardly considering subjective experience factors of users. Hence, it cannot be extensively applied.

#### Combination of subjective and objective methods

The combination of subjective and objective method has the advantages of both subjective evaluation and objective evaluation, taking the characteristics of users’ subjective feelings and the real-time demand for instantaneity of objective evaluation into account concurrently. This method could maintain user’s perception accurately, but it needs enough supporting data as well as model establishment and training. The existing researches only care one aspect or some aspects of different service types, and a unified model is lacked. Therefore, it is urgent to propose a method to support the evaluation of various services.

The current research achievements of end-to-end QoE evaluation are concentrated on the direct evaluation of media service at the terminal side, which is direct and effective. Nevertheless, there still are some existing problems (Jingjing and Nirwan 2011; Brooks 2010).

Firstly, the evaluation error is too large (Msakni and Yousef 2012). End-to-end QoE evaluation system requires higher computing capacity of terminal devices and tends to be easily affected by the end-to-end network environment, which will lead to inaccurate service QoE evaluation results and misleading.

Secondly, the extensibility is poor (Zhou et al. 2013). With the emergence of various new applications, it has become difficult to realize the update and maintenance of services at the client side, as the evaluation model is designed based on related service in accordance with end-to-end service QoE evaluation method.

Thirdly, there is a lack of coordination mechanism. End-to-end QoE evaluation system only cares about media interaction parameter changes at the client-side nodes, lacking enough consideration of the collaborative problem at transmission relay nodes or in other media transmission paths, which will reduce the accuracy of service QoE values.

### Typical application scenarios

**Fig. 1:**
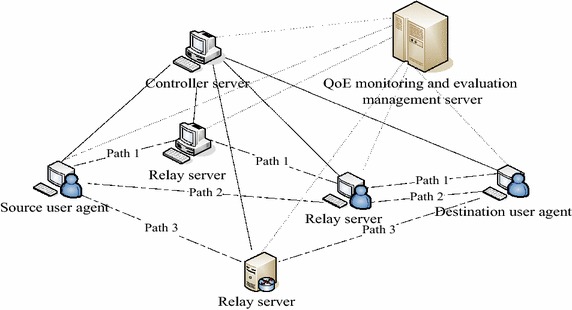
Collaborative evaluation system for service quality of experience

Multipath transmission technology can be realized on the following three network levels: (1) network layer, (2) transport layer, (3) application layer. At present, the transport layer of multipath transmission protocols, such as MPTCP (IETF 2011), requires communication endpoints to be a multihomed host, and needs to update the existing IP network protocol stack. The network layer multipath transmission technology mainly focuses on wireless transmission of video service routing. For example, a multipath transmission technology for Ad hoc network video service is proposed in (Gogate and Panwar 1994, 1999), which is to reduce the end-to-end delay for the selection of the optimal path. The application layer routing multipath transmission technology rebuilds the overlay network routing of this layer, retaining the existing network layer routing mechanism unchanged. Thus, the implementation is based on the application layer routing of multipath transmission. In previous work, a new QoE co-evaluation mechanism for multipath transmission has been proposed. QoE co-evaluation system consists of two parts, namely QoE monitoring and evaluation management server as well as QoE monitoring and evaluation management client. This paper is based on the application layer multipath transmission technology to study service QoE evaluation mechanism. Multipath transmission system relay framework based on application layer (Lei 2014a, b, c) mainly contains three parts, as shown in Fig. 1, i.e. controller server, relay server, and user agent. They act as QoE monitoring and evaluation management client to provide their own QoS parameters, user subjective parameters, and node position message for QoE monitoring and evaluation management server and collaborate with it to complete service QoE score. In QoE co-evaluation system, the client is responsible for receiving monitoring request for server at the node positions, and feedback IP address, QoS parameter message and user subjective parameter message to the server, which will score and manage these messages.

QoE co-evaluation mechanism is designed to realize QoE calculation mode of the server side. Therefore, QoE evaluation method is particularly important, which needs client parameters, media database coordination, monitoring and management module administration. In this paper, a kind of QoE co-evaluation method based on fuzzy clustering heuristic algorithm is proposed to carry out QoE evaluation management at the server side.

## QoE evaluation method design

Abandoning the decentralized management modes at the client of end-to-end QoE evaluation system, QoE co-evaluation method based on fuzzy clustering algorithm adopts centralized management modes to unify the service evaluation management process of the server side. With this method, the server side collects media parameters and user information of client feedback information in the evaluation process. According to the network status, “big data” processing method is utilized for the evaluation model to do fuzzy clustering analysis. Finally, service QoE evaluation score is predicted in accord with heuristic rule, and management information is feedback to the client to guide media transmission path selection and payload distribution, so as to complete QoE co-evaluation.

Most existing end-to-end service QoE evaluation methods are derived from the evaluation methods based on linear evaluation model. These methods depend on the majority of evaluation information composition rules of setting the model and linear model with great proximity. This process is called qualitative data analysis. The results of qualitative data analysis are usually restricted in a certain range. However, not all evaluation information can be evaluated accurately, which falls on the qualitative model, compared with the evaluation on the presence of the actual deviation value. The advantages of clustering model are intuitive, and the conclusion is simple. Fuzzy clustering analysis is to establish fuzzy similar relation based on the characteristics, intimacy, and similarity, realizing clustering analysis method for objective things. The QoE evaluation method of fuzzy clustering heuristic algorithm begins with the service characteristics. For each service periodic evaluation parameter, accurate service score should be given, the deviation degree of evaluation reduced, and the precision of calculation improved.

QoE co-evaluation method based on fuzzy clustering heuristic algorithm uses the existing “big data” processing technology (Lin et al. 2012; Kumar 2013; Shan et al. 2011), knowledge discovery (Hall et al. 2009), artificial intelligence heuristic method (Bhaskara 2011), and fuzzy comprehensive evaluation method act as the theoretical prototype to realize the evaluation method that meets the multipath transmission QoE co-evaluation model. Big data analysis can be realized with the presence of huge amounts of data in the law. Service evaluation from the perspective of big data analysis can get the characteristics of service convergence and self-similarity, and establish an empirical model through data analysis. Based on the empirical model of big data, this paper is to evaluate periodic transmission media service. For the newly established media session, the evaluation and guidance information can be directly extracted from database, such as transmission path and payload distribution. In this way, a lot of computing time can be saved. Details explanation will be given below.

### Evaluation rules design

In view of the typical scenarios of multipath transmission QoE co-evaluation model, a QoE co-evaluation method based on fuzzy clustering heuristic algorithm is proposed. The model is a kind of experience heuristic QoE evaluation method based on knowledge discovery, artificial intelligence and fuzzy mathematics to analyze the network performance status from the characteristics. This method discards the previous end-to-end linear quantization method and mode, and takes user’s own experience as the starting point, big data processing of media database as the theoretical foundation, as well as knowledge discovery, artificial intelligence and fuzzy mathematics as the theoretical background, to comprehensively analyze evaluation of information service.

The design of QoE co-evaluation method is divided into the following two steps: (1) Analysis of network performance perception and parameter index. (2) Design of service evaluation rules, which will be discussed in detail in the following parts.

#### Analysis of network performance perception and parameter

So far, the scale of network has been getting increasingly large, the booming information technology has become more and more complex, and the new applications of multimedia technologies have been increasing. Therefore, user satisfaction has become the focus of people’s attention to network servers, which has put forward higher requirements for QoS of network transmission. Backbone network signal analysis based on network flow analysis can better control the performance of the network, QoS index and access management. At present, the research of network flow is mainly aimed at the characteristics of network traffic and related metrics. For example, self-similarity is proposed by Leland in the early 90s (Leland et al. 1994; Paxson and Floyd 1995). Karagiannis et al. revealed that backbone link traffic of the Tier1 ISP discovered in the analysis on high bandwidth and high aggregate link traffic flow in sub-second scale satisfies the approximate Poisson process (Karagiannis 2004) in the early 21st century. These studies have triggered again people’s new thinking of network flow characteristics and modeling.

In the existing researches, statistical analysis is mainly conducted from the following two aspects, namely self-similarity under large time scale and multi-fractal characteristics under small time scale. No matter what method of research is utilized, their own network flow rules would be found out. Network flow analysis has a certain effect on the QoS parameters of network transmission. With the development of new applications, people have had a higher network utilization rate. From the perspective of practical application, the network flow is random. Consequently, it can be regarded as a random process. Network flow presents four kinds of states, namely excellent, good, medium, and bad states. Among them, “excellent” means that the network state is very good, and the network transmission is smooth. This state will not be affected by random bursts, or take on any congestion and other bad conditions. “Good” indicates that the network can fully guarantee the transmission media applications for service transmission and required parameter indexes, which may be subject to the influence of random bursts. “Medium” means that the network can provide QoS parameters which can be controlled in a good transmission range of media service. Nonetheless, there may be situations such as congestion and network queuing. “Bad” means that it cannot provide QoS guarantee for the transport service.

In order to obtain network flow performance information, the server uses ADC clock frequency sampling for the detection, to timely feedback network status information. Network flow classification is based on the network flow detection response message. That is to say, it can be expressed as below:

3 $${\text{NetFlow}} = \forall \left\{ {\text{excellent, good, medium, bad}} \right\}$$

**Fig. 2:**
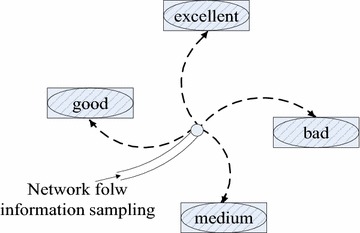
Network flow distribution of stochastic process

Network flow shows an irregular random condition. When the transmission signals of media services arrive, the flow status of classified network is extracted according to Formula (3), as shown in Fig. 2.

There is a randomness of network flow, based on which network flow status is divided into four parts. The definition only takes the characteristics of the network into consideration, abandoning small and large time scale analysis technology. The burstiness of network causes the result that there is no correlation among the states. In other words, they exist independently. A sudden flood and other emergency situations may result in the state transition of the network.

As the randomness of network flow state affects QoS parameters in media transmission, the process of network flow state analysis is as follows.

1. The server extracts the network flow status information from the classified media database server, and sets detailed QoS parameters for each corresponding state.
2. The server collects network flow information and keeps the information in media database.
3. The server extracts QoS parameter threshold range of the corresponding service transport according to network flow status information to meet the service QoE evaluation requirements.

The QoS parameters are affected by the network flow, which exhibit different characteristics and parameter threshold value ranges in different states. Parameters such as delay between two communication hosts may be defined as fixed delays, namely multi-hop transmission path processing delay and queuing delay (Mao et al. 2005; Mao 2005). With the increase in network speeds, the fixed delay has become comparable to the queuing delay. For the delay of the QoE parameters required for evaluation, the fixed delay is extracted from the receiver host in this paper. For streaming media service HD video stream with an “excellent” network state, the detected periodic time delay values of transmission are shown in Table 1.

**Table 1:** Detected periodic delay value for HD streaming media

Detection period	Delay values (s)
1	0.0003
2	0.0006
3	0.0001
4	0.0011
5	0.0025
6	0.0007
7	0.0013
8	0.0052
9	0.0011
10	0.0019
11	0.0035
12	0.0009
13	0.0010
14	0.0018
15	0.0014
16	0.0008
17	0.0033
18	0.0041
19	0.0022
20	0.0013
21	0.0052
22	0.0016
23	0.0010

**Fig. 3:**
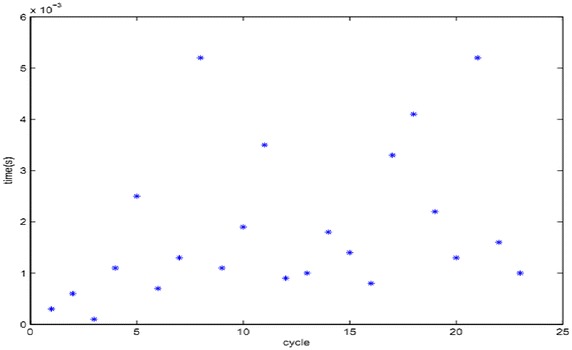
Delay parameter distribution of video streaming media

**Fig. 4:**
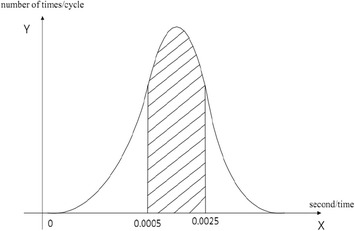
Normal distribution law of tested service parameters

For the design of QoS parameter model, two hypotheses can be made: (1) The detected parameters information is reasonable. (2) Parameter distribution is continuous. In media database, big data parameter information comes from the data obtained in client-side periodic detection fed back to QoE monitoring and evaluation management server. The parameters of the server are analyzed and evaluated, and QoE score is calculated. Finally, parameters and score information are stored in media database as big data source. As shown in Fig. 3, it can be seen that the distribution of parameters of the tested service has a certain degree of regularity. The parameter distribution obtained by combining parameter distribution map and database big data extraction processing meet the normal distribution in different time ranges. As shown in Fig. 4, probability distributions of the measured data onto the time interval of 0.0005–0.0025 s, which realizes the normal distribution in the interval range.

According to the different needs of service types, the value ranges of network transmission QoS parameters are not identical in different time ranges, which basically take on a normal distribution. Similarly, other indicators of QoS parameters such as jitter, packet loss, bandwidth, and load distribution background also meet normal probability density function. Among them, there are many factors that affect the distribution of parameters, such as multi-path transmission control, service demand, and network flow. The server may be able to provide users with better transmission performance through the management control of multipath transmission services, making network transmission parameters present corresponding distributions.

However, simply and randomly dividing network traffic into four grades of state is too coarse, which is not rigorous in different contexts or session times. Thus, network transmission parameters are specified for each random state. For example, the server is set to make certain specific adjustments when the network QoS parameters appear to float within a certain time period. Here, a detailed set of QoS parameter indicators in service QoE evaluation is arranged, covering delay, jitter, packet loss rate, and background payload bandwidth. In each random state, each parameter indicator has a certain level of granularity, and each level also contain a certain range of parameters. For instance, when delay parameter information is “good” in a random network status, parameter classification of the server side can be obtained, as shown in Table 2.

**Table 2:** Classification of delay parameters under a “good” network state

Grade	Range (ms)	Network state
1	(0, 50)	Good
2	(50, 100)	Good
3	(100, 150)	Good
4	(150, 300)	Good
5	(300, 400)	Good

According to the features of service, the server will directly feed network redirection information back and reselect the media transport path, when the critical value is lower than the minimum threshold.

Similarly, for other parameters in different network status or the same network state, the corresponding levels of classification and parameter range are not the same, which will not be listed in this paper. Therefore, under the state of network, network parameter level setting and the scope of classification provide a more detailed reference for service QoE evaluation.

#### Design of service evaluation rules

Condition attribute set C = {a_1_, a_2_, a_3_, a_4_, a_5_, a_6_}, and decision set P = {d_1_, d_2_}. QoE co-evaluation based on fuzzy clustering heuristic algorithm is described as follows,

*Input* condition attribute set C = {a_1_, a_2_, a_3_, a_4_, a_5_, a_6_}.

*Output* decision set P = {d_1_, d_2_}.

a_1_–a_4_ in C represent path measurement indexes of tested network (QoS parameters of network transmission, including delay, jitter, packet loss rate, bandwidth parameter information, which can be obtained through the detection made by the server side by sending probe data packets to the client device directly), involving service features (network transmission service type information), which can be divided into four categories according to 3GPP specifications, namely session class, streaming class, interactive class and background class. a_5_ and a_6_ respectively represent users’ subjective expectation attribute information (including the users’ subjective expectation threshold and service tariff standard, which can be obtained through the detection made by the server side by sending detection data packets to the client device directly) and node location information. d_1_ and d_2_ in P respectively represent assigned weight of path and predicted QoE threshold.

*Step 1* Determine network statuses according to feedback information of network flow detection.

*Step 2* Locate media database in accordance with the parameters information in Set C, and the historical service QoE score range by searching the database for other parameters information in the light of the rules of depth first search rule.

*Step 3* If there are historical score data in media database, score the service based on the historical evaluation. Otherwise, go to Step 4, recalculate service score and store it in media database.

*Step 4* Transmission media service QoE score calculation of can be carried out in accordance with the following rules.

*Step 4-1* End users can directly give service experience score. If so, the corresponding transmission service indicators, such as network transmission QoS parameter information, media information, node position information, relay routing and relay node information shall be recorded in the database as the historical data.

*Step 4-2* Otherwise, the score shall be calculated. In this paper, the design of the QoE score is accomplished by employing intelligent inference machine method to avoid the constraint by linear method, and directly reasoning on the basis of the parameter indexes.

*Step 4-2-1* List all the specification information for service evaluation.

*Step 4-2-2* Analyze the service type, and identify the most critical one or more index parameters that affect service evaluation.

*Step 4-2-3* Conduct classification and piecewise analyses on the parameters according to the possibility of score, and achieve parameter indexes at corresponding stages.

*Step 4-2-4* Match the existing service transmission parameters, and get the score information.

*Step 5* Feedback to the Client P.

The first scoring rule of service QoE is stored in media database. When the media database doesn’t have historical evaluation data, the scoring rules are to be utilized.

Before establishing the media session, the server establishes QoE score service back-up to record information including IP addresses, port number of session, transmission service type, relay transmission paths and node information. After media session is established, the server sends probe data packets of QoE evaluation parameters to the client and waits for it to feedback parameter information. After the client feeds back the evaluation parameter information, the server side starts to carry on service QoE evaluation and feeds back client decision information after QoE evaluation.

Following the big data analysis principle, QoE evaluation based on fuzzy clustering heuristic algorithm can obtain predictive score value and management information eventually, after the service analysis according to fuzzy matching and heuristic rules.

### Heuristic service quality evaluation

Heuristic rule evaluation has a low-complexity algorithm which can be used to evaluate the dynamic and complex large-scale parameters. After the server has determined certain evaluation rules, corresponding rules are stored in rule bank. With more and more media database evaluation information, evaluation rules are more prefect, and evaluate performance will become better and better. At the same time, big data information can correct evaluation rules to make QoE more and more accurate.

Heuristic service quality evaluation is mainly reflected in the process of QoE evaluation, and the server provides a set of rules to meet users’ demands. When two clients are in the media session, the server records and starts the service evaluation program, and carries out heuristic evaluation according to the periodic parameters information fed back by the client.

Under certain constraint conditions, decomposition in allusion to an indicator can be completed judging by service features in heuristic service quality evaluation, such as QoS parameters of network transmission which can extract the key influencing elements of service types to evaluate so as to obtain the best service score. The factors affecting service QoE evaluation include service type, network transmission QoS parameters, user subjective expectations, node location, and so on. These are constraints, and some of them have to meet the requirements for QoS parameters, such as network transmission, which is the basic need of service transfer. Different rules have different implementation schemes according to the constraint condition information. In consequence, heuristic evaluation rules are divided into two conditions as follows.

1. Policy-oriented implementation rules. It is similar to the algorithmic game theory. After the client feeds back to the server the necessary evaluation parameters, which just fit into the implementation rules, and the media database has these parameters opportunely, QoE scoring and management shall be executed. The evaluation implementation process is called as policy implementation rules.
2. Thinking-oriented implementation rules. When there is no evaluation information the corresponding to the needed evaluation parameters information fed back by the client to the server, the first evaluation rule shall be used for the evaluation, and inserted into the media database as the basic big data information.

Service QoE evaluation information is divided into two parts. One part contains the objective parameters, namely transmission network QoS parameter index and relay node parameter information. The other part covers the users’ subjective parameter information. In the process of service evaluation, two factors should be considered. For the evaluation score, MOS score mapping relationship is shown in Table 3.

**Table 3:** MOS mapping of QoE evaluation

User expectations	Network parameters	MOS values	Policy decisions
U_value_ = 5	N_value_ = 5	5	1
U_value_ = 4/5	N_value_ = 4	4	1
U_value_ = 4	N_value_ = 4/5	4	1
U_value_ = 3/4/5	N_value_ = 3	3	2/3
U_value_ = 3	N_value_ = 3/4/5	3	2/3
U_value_ = 2/3/4/5	N_value_ = 2	2	2/3
U_value_ = 2	N_value_ = 2/3/4/5	2	2/3
U_value_ = 1/2/3/4/5	N_value_ = 1	1	2/3
U_value_ = 1	N_value_ = 1/2/3/4/5	1	2/3

Among them, the range of U_value_ of user expectations is 1– 5. 1, which indicates that the user score is the worst and completely unacceptable. The number 5 suggests that the user experience is very good and fully in line with their expectations while the media service experience is satisfying. For network QoS parameters, one can refer to Table 4 which shows the values of different parameters in each network state. Parameter values of D_Grade_, J_Grade_, P_Grade_, and B_Grade_ are given in Table 2, which shows the range of examples in detail. N_value_ value method is used for Buckets effect. If one of the previous parameters is lower compared with the others, N_value_ should have the lowest value. Similarly, MOS mapping value is min {U_value_, N_value_} in the MOS mapping table. Policy decisions represent the guidance of media transmission which is given by QoE monitoring and evaluation management server according to service evaluation score and network transmission QoS index. Policy decisions can be categorized into three kinds, using the numbers 1, 2 and 3 to denote policy implementation plans. The relevant policy decisions are described as follows.

**Table 4:** Network parameters reference

Network state	Delay	Jitter	Packet loss rate	Bandwidth	N value
NetFlow	D_Grade_	J_Grade_	P_Grade_	B_Grade_	N value

Service QoE evaluation score is to provide services for media transmission control. In the process of media transmission, there are some new technologies, such as media streaming payload distribution on the transport path (Ning et al. 2012; Song et al. 2012). Service QoE evaluation algorithm will give guidance to meeting the transport needs of media. In this paper, policy decision value 1 means that the server will directly give user experience score of media service, and feed it back to the client. Policy decision value 2 signifies network redirection, i.e. adjust of the media transport payload or paths. Policy decision value 3 suggests that the transmission of the media service should be stopped.

Policy decisions are the ultimate goal of media transmission control in service QoE evaluation, which can help satisfy the user experience quality requirements and meet the demand of media transmission. In media transmission, streaming payload is closely related to the background payload bandwidth. In the allocation of transmission, the server can be assigned based on the transmission strategy of “from each according to his ability”. That is to say, each path payload distribution ratio is equal to the background payload bandwidth value divided by the total sum value of all transport paths’ background payload bandwidth, as shown in Formula (4).

4 $$L_{i} = \left\lfloor {\frac{{B_{i} }}{{\mathop \sum \nolimits_{j = 0}^{num - 1} B_{j} }}} \right\rfloor$$

wherein, *L*_*i*_ is the transmission payload distribution ration of path *i*. *B*_*i*_ is the background payload bandwidth of path *i*. *j* represents the transmission path, and its value is [0, *num*-1]. *num* denotes the number of transmission paths.

The calculation of the parameter value of background payload bandwidth can be achieved using a sub-path transmission stream and the original background idle bandwidth ratio method. When the payload bandwidth ratio is between (0, 30 %), the transmission state is excellent. When it is between (30, 70 %), this situation can meet the transmission requirements. Nevertheless, network congestion may emerge at any time. When it is higher than 70 %, the server will automatically consider the transmission media to have consumed the payload bandwidth of path, which does not meet the demand. Under this condition, it needs to make payload distribution adjustments according to Formula (4). Policy decision value 2 in Table 3 indicates that the server needs to make payload distribution adjustments according to Formula (4), or redirect network to choose other transport paths. Policy decision value 3 suggests that none of the transmission schemes can meet the demand of media transmission, which should be stopped consequently.

### Fuzzy clustering analysis

Fuzzy clustering analysis is a necessary step in the process of big data processing in media database. The server handles client feedback evaluation parameters, and eliminates redundancy to find similar evaluation information set, process data in accordance with the rules of knowledge discovery, and finally gets the excellent, good, medium, passing and poor evaluation results.

Big data information in media database is obtained through a massive computational analysis following evaluation rules. The composition of big data into media database includes several aspects, namely service type of media transmission, network transmission QoS parameters, user subjective factor, node location information, and service score information. The server collects and stores the required parameters of each evaluation through media service evaluation, which will be put into the media database as the big data source simultaneously.

Currently, the study of big data has become a hot spot. High-capacity, high-production rate, and varied information values need to re-processed to ensure the accuracy of the judgments and decisions on the basis of big data. The existing studies of big data are mainly focused on how to store, process, analyze and manage big data. Nonetheless, this paper mainly emphasizes data processing technology for the application of big data in media database, including data storage technology, data mining technology based on data mining, large data processing technology based on query, data processing technology based on knowledge discovery in data mining, which are applied to analyze and process data, and obtain the effective data, so as to carry out fuzzy clustering analysis.

Fuzzy clustering analysis uses “thinking-oriented implementation rules” in “Heuristic service quality evaluation” section according to “big data” in media data. When the media database does not have the required information and scoring information on evaluation, the server will put media transmission detection data and evaluation data information into the database as big data source. Finally, the server will implement the rules of fuzzy clustering heuristic algorithm analysis after there is a satisfying threshold amount of data in media database.

In fuzzy clustering analysis, the server uses system clustering method to process data. With this method, all the client’s feedback parameters are regarded as a large amount of sample information, and the evaluation is a data matrix, abstractly:

5 $${\text{x}} = ({\text{x}}_{\text{ij}} )_{{{\text{n}} \times {\text{m}}}} = \left[ {\begin{array}{*{20}c} {{\text{x}}_{11} } & \cdots & {{\text{x}}_{{1{\text{m}}}} } \\ \vdots & \ddots & \vdots \\ {{\text{x}}_{{{\text{n}}1}} } & \cdots & {{\text{x}}_{\text{nm}} } \\ \end{array} } \right]$$

wherein, *n* is the number of samples in the database which is close to that of evaluation parameters, *m* is the variable number of factors affecting the application of evaluation. Therefore, all the parameter information values of the *ith* sample can be obtained through:

6 $$({\text{x}}_{{{\text{i}}1}} ,{\text{x}}_{{{\text{i}}2}} , \ldots ,{\text{x}}_{\text{im}} )^{\text{T}} , \quad{\text{i}} = 1,2, \ldots {\text{n}}$$

Then, the values of sample service information are inserted into the extracted matrix, i.e.:

7 $${\text{X}} = \left( {{\text{x}}_{\text{ij}} } \right)_{{{\text({n}} + 1) \times {\text{m}}}} = \left[ {\begin{array}{*{20}c} {{\text{x}}_{11} } & \ldots & {{\text{x }}_{{1{\text{m}}}} } \\ {\begin{array}{*{20}c} \vdots \\ {{\text{x}}_{{{\text{n}}1}} } \\ {{\text{x}}_{{\left( {{\text{n}} + 1} \right)1}} } \\ \end{array} } & {\begin{array}{*{20}c} \vdots \\ \ldots \\ \ldots \\ \end{array} } & {\begin{array}{*{20}c} \vdots \\ {{\text{x}}_{\text{nm}} } \\ {{\text{x}}_{{\left( {{\text{n}} + 1} \right){\text{m}}}} } \\ \end{array} } \\ \end{array} } \right]$$

Euclidean distance calculation method is utilized to calculate the distance between the historical data sample and evaluating sample. The formula is as follow:

8 $${{\text{d}}_{{\text{i}}({\text{n}} + 1)}} = {\left[ {\sum\limits_{{\text{k}} = 1}^{\text{m}} {{{({{\text{x}}_{{\text{ik}}}} - {{\text{x}}_{({\text{n}} + 1){\text{k}}}})}^2}} } \right]^{1/2}},\quad{\text{i}} \in (1,{\text{n}})$$

According to the Euclidean distance calculation method, all the distances between evaluating samples of service QoE and the other samples are obtained. Finally, the two samples with the shortest distance are merged into one category to complete QoE score.

QoE score evaluation is utilized to manage nodes and media transmission paths. When the QoE score is lower than the default value, the server will send a management message to the client to adjust media transmission paths, so as to control media transmission.

## Implementation of QoE evaluation method

With QoE co-evaluation method based on fuzzy clustering heuristic algorithm, relevant parameter information under network states is analyzed according to big data processing, and then, score prediction calculation is achieved in accordance with heuristic rules. Finally, the analysis on the obtained evaluation score and the management of information are realized based on fuzzy clustering analysis. QoE evaluation cannot give a detailed service score of QoS parameters of network transmission. As a result, to choose a good prediction method is the focus of the scoring rules. In the past, QoE evaluation system simply relied on linear rules to give the scoring rules, which violates the characteristics of randomness and burstiness of network signals as not all the state score information can be covered. This paper proposes a method that combines data processing modes of server media database and heuristic evaluation rules to obtain service evaluation scores and management information in the fuzzy clustering analysis based on network rules. The method is divided into the following steps.

1. Data capturing. The server captures network state data information and determines the state of network flow according to collected information.
2. Extraction of QoS parameters of network transmission. QoS parameters are matched according to network flow state information in order to remove a great deal of irrelevant information in the process of QoE score.
3. Depth-first parameter searching. Depth-first searching method for big data in the database is adopted to process QoE evaluation parameters depending on the service type of the server, with the sensitive parameters as the initial conditions. That is to say, one of the parameters is taken as a starting point for traverse search, which will not stop until all the required evaluation parameters are found out. Then, the historical evaluation information on database is obtained. The historical data are stored in media database in a linear way, including service type, delay, jitter, packet loss rate, and bandwidth, relay node types, users’ expectations, as well as service score.
4. Prediction of score based on heuristic rules. Network transmission QoS parameter information is processed in the first three steps, and then user subjective parameters and node location information are predicted on the basis of heuristic rules.
5. Fuzzy clustering analysis is made on the database score result information set obtained based on heuristic rules. The final score and management information are obtained.

**Fig. 5:**
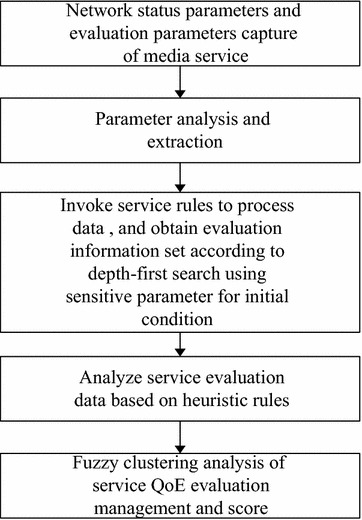
Analysis based on fuzzy clustering heuristic QoE evaluation rules

In summary, the analysis process of QoE evaluation based on fuzzy clustering heuristic rules can be described in Fig. 5.

QoE monitoring and evaluation management server is the essential for QoE co-evaluation based on fuzzy clustering heuristic algorithm and other clients’ collaboration, which can be achieved by using centralized management framework. In the process of service QoE co-evaluation, QoE monitoring and evaluation management server periodically gathers service evaluation parameters information, which enables media service at the server side to evaluate periodically. At the same time, the server must gather and store corresponding evaluation score into the media database as history information. The evaluation information includes all the required parameters of evaluation, which can serve as the reference for media evaluation fuzzy matching and score.

In media service transmission, the server will give the network redirection policy decisions to reselect relay paths to media transmission control, when the network status changes significantly, such as the situation that one path cannot carry on the transmission due to network congestion in multiple paths. However, not all situations require network redirection. When a small change occurs in the network, the server side gives out the decision information to redirect the network, which will cause great pressure on the calculation of the server. Therefore, QoE monitoring and evaluation management server carries out fuzzy clustering analysis in media service score calculation at the beginning of design. That is to say, service score and policy decision of media transmission maintain the same in case of a very small change in network transmission QoS parameters. Among them, the factor that hinders server recalculation is set as blocking factor *ɛ*. In media transmission, there is a certain range of*ɛ*. The range setting is shown in Table 5.

**Table 5:** Range setting of blocking factor among network QoS parameters

*ɛ*	Range
*ɛ*_*D*_	10 ms
*ɛ*_*J*_	5 ms
*ɛ*_*P*_	0.5 %
*ɛ*_*B*_	10 %

Herein, *ɛ*_*D*_ represents the limited scope of delay, *ɛ*_*J*_ that of jitter, *ɛ*_*P*_ that of packet loss rate, and *ɛ*_*B*_ that of background payload bandwidth ratio. When these parameters change within the range of blocking factors, the network will no longer redirect and restore calculation. QoE monitoring and evaluation management server will give out the information of original decisions according to the original score.

In the process of service QoE evaluation, QoE monitoring and evaluation management server using Remote Network Monitoring (RMON) protocol periodically reports statistical service evaluation parameters information. RMON sampling period range is set between 0–1800 s, and the default value is 1800 s. The existing technologies of playing mostly use watching and buffering method, such as You Tube and iQIYI. Buffering time is usually 50 s. Of course, these techniques can also be utilized to buffer all the media methods for user experience. If the default value is set at 1800 s, a lot of videos may not be periodically evaluated and stopped, which cannot meet the needs of user experience and media transmission control. Therefore, the sampling period is chosen to be 50 s in the design method proposed in this paper. Every 50 s is a period for media transmission service QoE evaluation.

From the perspective of big data analysis, the realization process of QoE evaluation method is as follows.

1. Data collection. The server side extracts parameters of service QoE evaluation.
2. Data access. The history evaluation information of server composes the relational database. The parameters information of the periodical evaluation on transmission service is carried out in the media database, and the parameters are extracted and matched.
3. Infrastructure. Cloud storage technology can be widely used for storing big data of historical evaluation information.
4. Statistical analysis. Fuzzy clustering heuristic algorithm is employed for data processing.
5. Data processing. Information stored in the database serves as history information for the evaluation, including network QoS parameters of the media session, information of nodes and paths, user subjective expectations, the result of evaluation and policy decisions.
6. Model prediction. Evaluation score of the next cycle or media service session and media transmission policy plan are predicted.

## Results and discussion

### Simulation and verification

As a discrete event simulation tool, OMNeT++ can be used to simulate any discrete event system, including simulation communication protocols, computer networks, distributed systems and so on. The simulation is based on multipath transmission model, and network probing packets are transmitted from the server side to detect network flow state. Meanwhile, service QoE is evaluated according to QoE evaluation parameters. HD video streaming media transmission service acts as the emulational test service of QoE co-evaluation method simulation based on fuzzy clustering heuristic algorithm. In the simulation, the server side needs to classify network state according to media databases, and detect network transmission QoS parameters.

In HD video streaming service transmission simulation, the simulation platform involves several parts, including two user agents (source user agent User1, and destination user agent User 2), four relay servers (Relayser 1, Rlayser 2, Relayser 3 and Relayser 4), one controller server (controller), and one QoE monitoring and evaluation management server (qoeser). Each functional entity is connected by routers, and network QoS parameters can be set according to different simulation requirements, such as network bandwidth, packet loss rate and transmission delay. The carefully arranged QoE monitoring and evaluation management server can give play to its QoE score calculation function in its internal QoE score calculation module after receiving the service QoE evaluation parameter information sent by the agent. At the same time, media transmission paths can be assigned by the controller server according to allocation rules.

**Fig. 6:**
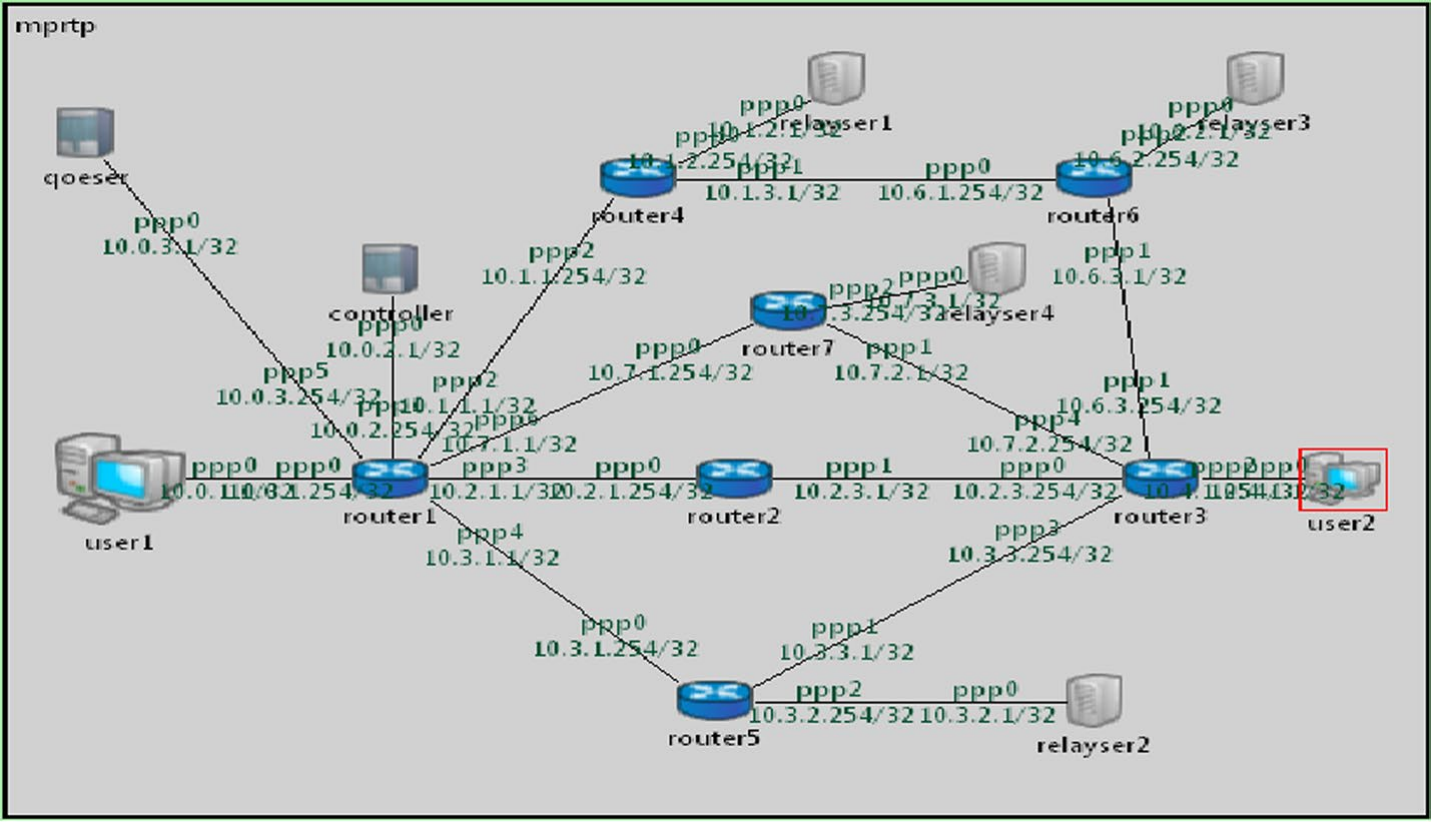
Multipath relay transmission network deployment

In multipath relay transmission, the number of relay nodes cannot exceed two. As a consequence, the deployment of multipath RTP (MPRTP) network, as shown in Fig. 6, represents the real environment of multipath relay transmission. Path 0: User 1- > User 2 (default route, with no relay node). Path 1: User 1- > Relayser 1- > Relayser 3- > User 2 (including two relay nodes). Path 2: User 1- > Relayser 2- > User 2 (including one relay node).

After the media service session is established, the server sends a probe packet to the client. When the client receives a request message, it feeds back the required parameter information to the server. QoE score is calculated and managed at the server side.

In the simulation, the media database is recorded as historical evaluation information. After the client periodically feeds back the service evaluation parameters information, QoE evaluation based on fuzzy clustering heuristic algorithm will be carried out in the following steps:

*Step 1*

Taking the third cycle evaluation in the process of media service evaluation for example, network state is analyzed and classified by the server according to network probe packet data, such as main network throughput, channel capacity, link utilization and response time. The high-quality network status, i.e. excellent state, is simulated.

*Step 2*

**Fig. 7:**
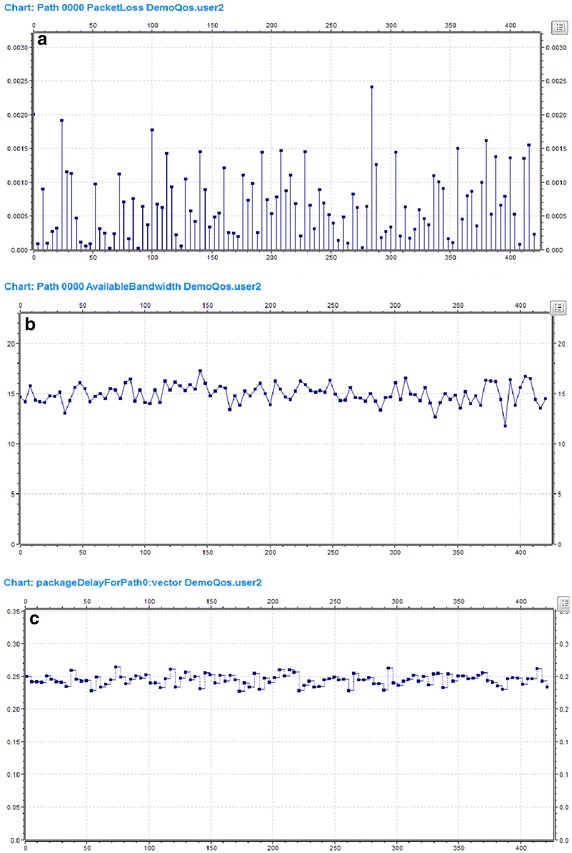
Network transmission parameters of Path 0. **a** Shows the periodic feedback information of packet loss rate parameter. **b** Denotes bandwidth parameter. **c** Represents delay parameter

The server extracts feedback parameters of the client, classifies them, and finally determines the parameters of evaluation. In the collection of evaluation parameters, the server needs to monitor each client’s parameter, and collects media transmission path parameters. As shown in Fig. 7, the server collects the parameters of Path 0 required for media transmission evaluation.

**Fig. 8:**
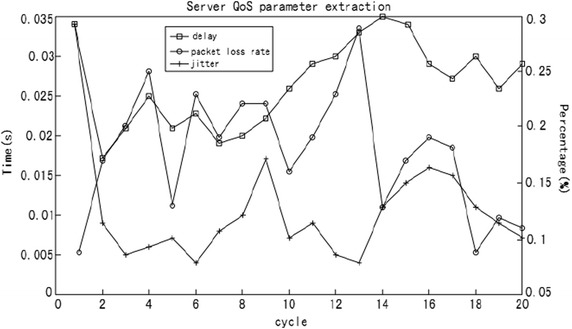
Network transmission QoS parameters extracted by the server

After extracting evaluation parameters information, all the parameters are classified to screen out the information for the QoE co-evaluation, as shown in Fig. 8.

*Step 3*

With one of the parameters as the starting point, such as delay, depth-first searching for historical data information in media database is carried out to select the historical evaluation data matching the database.

*Step 4*

The fuzzy matrix is built according to historical data (In the simulation, historical evaluation data is used to test sample data stored in media database. The data is obtained through massive client service detection and server score, to replace the big data sample information.). Each history evaluation information is regarded as a sample, as shown in the Formula (6). Historical data sample number is *n* which meets heuristic rules, with *m* sample properties. The *i*-*th* sample is expressed as follow:

$$({\text{x}}_{{{\text{i}}1}} ,{\text{x}}_{{{\text{i}}2}} , \ldots ,{\text{x}}_{\text{im}} )^{\text{T}} ,\quad {\text{i}} = 1,2, \ldots {\text{n}}$$

In the simulation, the value of *m* is 6. x_i1_ is the delay parameter of QoS parameters of network transmission, x_i2_ the jitter parameters of QoS parameters of network transmission, x_i3_ the packet loss rate of QoS parameters of network transmission, x_i4_ the payload bandwidth of transmission paths, and the value is 0, 1 and 2. 0 represents that network transmission bandwidth can meet service needs, but it is affected by the outbreak of network traffic at any time, while 1 denotes that network transmission bandwidth can meet service needs without any random situation, and 2 means network transmission bandwidth is lower than the critical value. x_i5_ indicates that the parameters of relay nodes location are 0, 1, and 2. Among them, 0 and 1 represent the communication between two parties in the same network and different networks respectively, and relay nodes are in one of the two parties’ networks. However, 2 means that relay nodes do not exist in both sides of the communication networks. x_i6_ refers to user subjective expectations parameters, and its value is one of 1, 2, 3, 4 and 5 given by users. Otherwise, it will be set at the default value, i.e. 4.

According to network transmission QoS parameter index, the historical data information set in the database that meets service transmission conditions is found out with depth-first searching method. The existing test data number that meets the requirement in the database is seven, and the fuzzy matrix is shown as follows.

$$\left[ {\begin{array}{*{20}c} {0.021} \quad & {0.005} \quad & {0.002}\quad & 0 \quad & 1 \quad & 3 \\ {0.021}\quad & {0.005} \quad & {0.002} \quad & 1 \quad & 0 \quad & 4 \\ {0.021} \quad & {0.005} \quad & {0.002}\quad & 0 \quad & 0 \quad & 5 \\ {0.021} \quad & {0.005} \quad & {0.002} \quad & 1 \quad & 2 \quad & 4 \\ {0.021} \quad & {0.005} \quad & {0.002} \quad & 0\quad & 1 \quad & 4 \\ {0.021} \quad & {0.005}\quad & {0.002} \quad & 0 \quad & 0 \quad & 4 \\ {0.021} \quad & {0.005}\quad & {0.002} \quad & 0 \quad & 0 \quad & 5 \\ \end{array} } \right]$$

Based on the law of fuzzy clustering analysis, the test periodic service evaluation information is merged into the fuzzy matrix, to obtain the new fuzzy matrix, which is shown below.

$$\left[ {\begin{array}{*{20}c} {0.021} \quad & {0.005} \quad & {0.002} \quad & 0 \quad & 1 \quad & 3 \\ {0.021} \quad & {0.005} \quad & {0.002} \quad & 1 \quad & 0 \quad & 4 \\ {0.021} \quad & {0.005}\quad & {0.002} \quad & 0 \quad & 0 \quad & 5 \\ {0.021} \quad & {0.005} \quad & {0.002} \quad & 1 \quad & 2 \quad & 4 \\ {0.021} \quad & {0.005} \quad & {0.002} \quad & 0 \quad & 1 \quad & 4 \\ {0.021} \quad & {0.005} \quad & {0.002} \quad & 0 \quad & 0 \quad & 4 \\ {0.021} \quad & {0.005} \quad & {0.002} \quad & 0 \quad & 0 \quad & 5 \\ {0.021} \quad & {0.005} \quad & {0.002} \quad & 0 \quad & 0 \quad & 4 \\ \end{array} } \right]$$

In fuzzy matrix clustering analysis, the server needs to calculate the distance between the evaluation sample and other historical data samples, and gets sample values and information with the shortest distance. Euclidean distance calculation method is adopted in this paper to calculate the distance between samples, as shown in Formula (8).

$${\text{d}}_{18} = 1.4142135624; {\text{d}}_{28} = 1; {\text{d}}_{38} = 1; {\text{d}}_{ 4 8} = 2. 2 3 60 6 7 9 7 7 5 ; {\text{ d}}_{58} = 1; {\text{d}}_{68} = 0; {\text{d}}_{78} = 1;$$

The shortest distance sample is x_6_, and the sample score is “good”.

*Step 5*

Fuzzy clustering analysis is made according to Step 4. The score samples and historical data samples x_6_ are clustered to get the clustering evaluation information, i.e. “good”, which can meet the needs of media transmission. Given this, evaluation management information is “to maintain the original transmission decision”, which will be sent to the client to manage the transmission control needs.

**Fig. 9:**
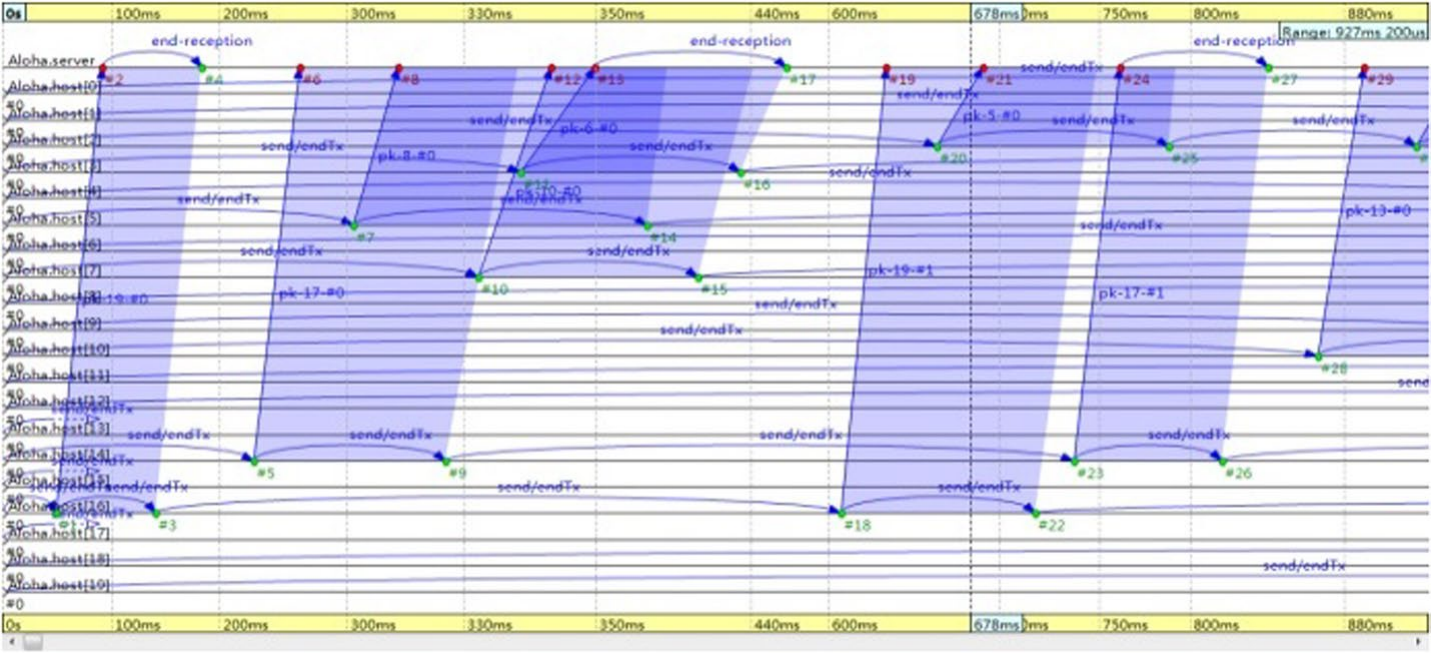
Overloaded network data output detected by the server

Due to the randomness of the network state, the simulation is in an “excellent” condition of network state to get service QoE evaluation data information. The random state of network can be simulated in the simulation, as shown in Fig. 9. In the test example, when network queuing, congestion, and other conditions emerge in transmission Path 0 because of overload, which result in affected media transmission, local network state is “poor” at this time.

After the emergence of overload phenomenon in local network, network transmission parameters detected at the relay nodes in the receipt of transmission media change. Once the server receives the receipt message, it conducts QoE co-evaluation according to QoE evaluation parameters and evaluation rules. The calculation process is the same as that presented above. Subsequently, the obtained QoE evaluation result is “medium”. At the same time, node information which does not satisfy the preset value of transmission parameters in the database is screened out, and transmission control management rules are presented. The transmission paths are readjusted according to the feedback parameters from the relay nodes and transmission paths. Then, backup path User 1- > Relayser 4- > User 2 is selected, so as to control media transmission and meets the needs of the users.

### Evaluation analysis

**Fig. 10:**
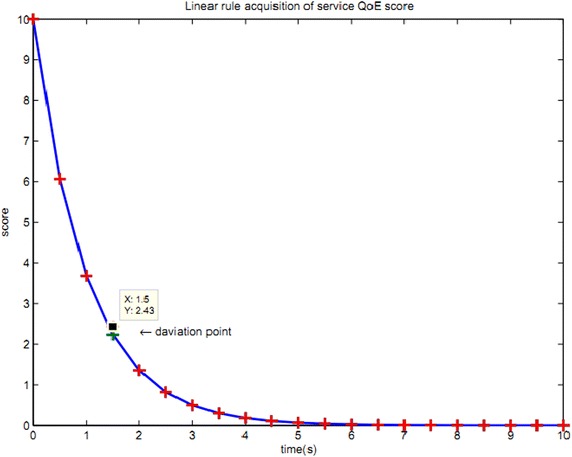
End-to-end QoE linear evaluation method

QoE co-evaluation method based on fuzzy clustering heuristic algorithm is achieved by big data processing, fuzzy clustering analysis on parameters and heuristic rules in media database at the server side. Compared with end-to-end QoE linear evaluation, the precision of user experience is improved, which is mainly reflected from the following three aspects:

1) Most of the existing QoE evaluation methods adopt linear models for evaluation, which has some limitations. Thus, it cannot generalize all the evaluation indicators effectively. Figure 10 shows a network segment of QoE model proposed in Literature (Chen et al. 2009).

From Fig. 10, it can be seen that end-to-end QoE linear evaluation method selects the parameter information about delay and jitter. With the increase in delay and jitter, QoE scores take on an inversely proportional exponential relationship. However, this kind of method can only be applicable to the majority of actual evaluation cases, excluding at least 5 % deviation situations, such as the deviation points in Fig. 10. Accurate evaluation information cannot be given after QoE linear evaluation. That is to say, there are some errors of service evaluation accuracy. End-to-end QoE evaluation system is mainly used to detect and evaluate media service at the client side. Nevertheless, this kind of QoE evaluation management model is not suitable for multipath transmission, in which the client cannot “see” transmission paths and relay node information to control the transmission microscopically. From the view of media transmission control, the centralized evaluation system QoE co-evaluation model shall be employed rather than end-to-end QoE evaluation system.

**Fig. 11:**
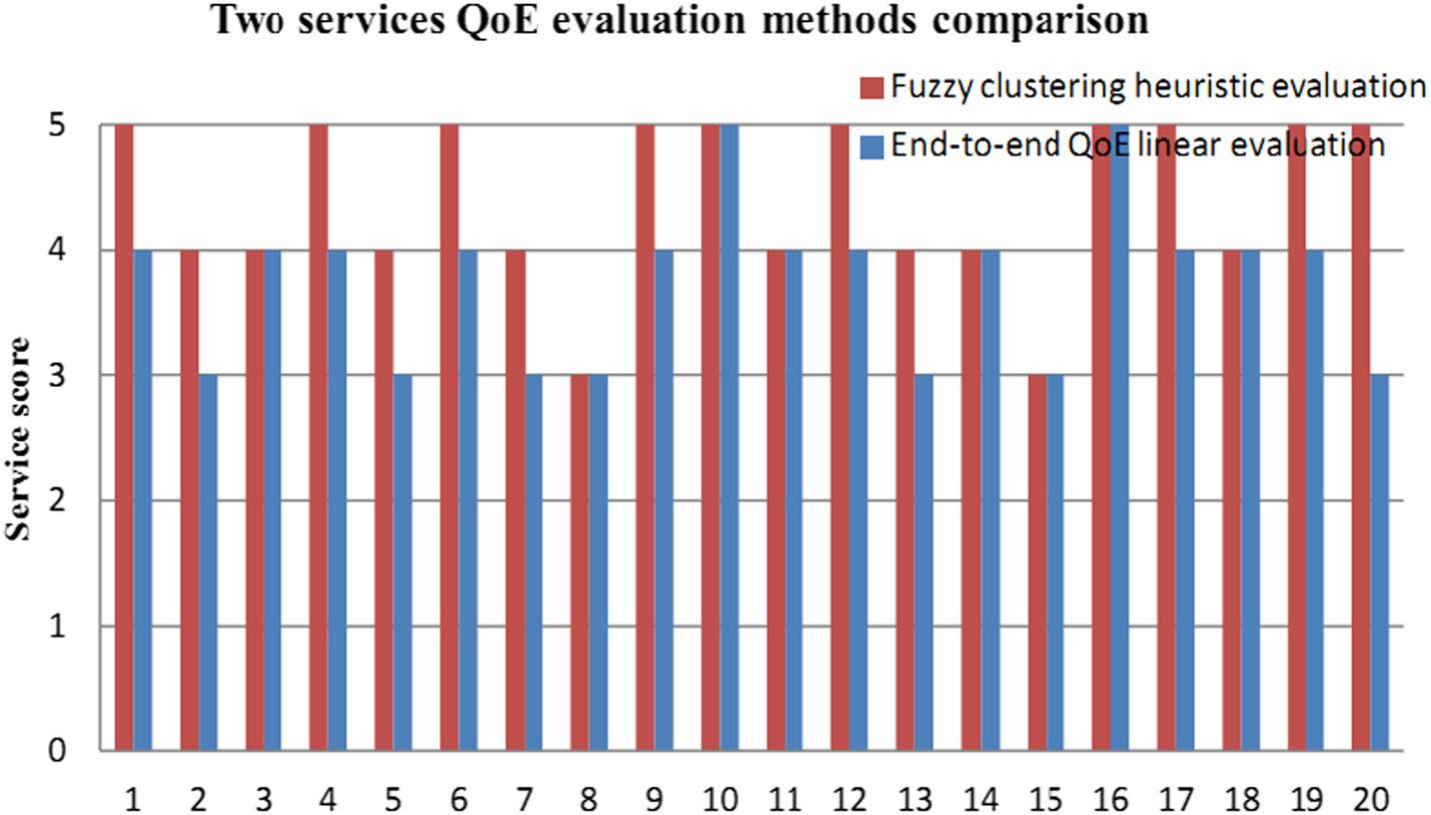
Comparative analysis of two kinds of service QoE evaluation method

For the control of media transmission QoE evaluation management, QoE co-evaluation model of multipath relay transmission can exercise macro control over paths and relay nodes. As new application may consume a large amount of bandwidth, the single path transmission could not meet transmission needs, which may even hinder media transmission. End-to-end QoE evaluation needs to maintain client service score information, which is difficult to improve with the emergence of new application. Therefore, the problem of separate maintenance exists at the client side. Besides, QoE linear evaluation at the client side is not accurate sometimes, misleading media transmission control. Figure 11 shows a comparison of QoE evaluation method based on fuzzy clustering heuristic algorithm and the evaluation method described in Literature (Chen et al. 2009).

Figure 11 presents the comparison of two kinds of service evaluation methods. The final results of QoE co-evaluation method based on fuzzy clustering heuristic algorithm are divided into five segments in order to ensure a clear comparison of two methods, namely excellent, good, medium, fair, and poor. According to MOS, the above evaluation results can be attributed to five scores, namely 5–1. Among them, 5 represents excellent and 1 indicates poor. End-to-end QoE linear evaluation method obtains score information with MOS that divides the results into five segments, which is the same as the case of multipath QoE co-evaluation method. As can be seen from the figure, there is a gap between the final service scores of the two evaluation methods.

For the service QoE evaluation analysis, the majority of researchers adopt BTL linear model analysis. In the BTL model method, the selection probability is a linear function as expected. The calculation of probability is achieved by dividing the expected utility of observed object by the total utility. However, the BTL analysis method is not suitable for the output score sequence of the existing two evaluation methods. Consequently, the standard deviation calculation method should be applied instead. As shown in Fig. 11, the evaluations of the two methods are not the same. To compare the two methods, the standard deviation method is utilized in this paper, as shown in Formula (9).

9 $$\sigma = \sqrt {\frac{1}{N}\sum\limits_{i = 1}^{N} {(x_{i} - \mu )^{2} } }$$

Herein, *σ*represents the standard deviation of service evaluation. *N* denotes the number of evaluation period. *x*_*i*_ means the score of each period in the process of evaluation. *μ* signifies the average value of the evaluation method. Table 6 shows the periodic evaluation score values of the two methods.

**Table 6:** Periodic score values of two evaluation methods

*x*_*i*_	5	4	4	5	4	5	4	3	5	5
	4	5	4	4	4	5	4	4	5	5
*x*_*j*_	5	3	4	4	3	4	3	3	4	5
	3	4	3	4	3	5	4	5	4	5

wherein, *x*_*i*_ represents the periodic score of fuzzy clustering heuristic QoE evaluation, and *x*_*j*_ indicates that of end-to-end linear QoE evaluation. According to the formula of standard deviation, the mean scores of the two evaluation methods are obtained.

$$\mu_{1} = \frac{1}{N}\sum\limits_{i = 1}^{N} {x_{i} } = 4.4$$

$$\mu_{2} = \frac{1}{N}\sum\limits_{j = 1}^{N} {x_{j} } = 3.9$$

On the basis of the existing conditions, the standard deviations of the two evaluation methods are acquired.

$$\sigma_{1} = \sqrt {\frac{1}{N}\sum\limits_{i = 1}^{N} {(x_{i} - \mu_{1} )^{2} } } = 0.58$$

$$\sigma_{2} = \sqrt {\frac{1}{N}\sum\limits_{j = 1}^{N} {(x_{j} - \mu_{2} )^{2} } } = 0.76$$

The statistical analyses of the two evaluation methods show that the mean score of the fuzzy clustering heuristic QoE co-evaluation is higher whereas its standard deviation is lower compared with end-to-end linear QoE evaluation. By referring to the expected value of user experience in media service transmission, QoE co-evaluation based on fuzzy clustering heuristic algorithm can better meet the demand of user experience. In addition, it is more accurate and reliable in term of the gap between the test value and the true value, namely standard deviation. In other words, the result of QoE co-evaluation based on fuzzy clustering heuristic algorithm is more close to user experience value in processes of user experience and media transmission control decision.

Although linear model is not used in Literature (Kumar 2013), the measurement parameters of video streaming QoE evaluation are only related to startup delay, average peak signal to noise ratio and buffer percentage. The simulation results are given in Figs. 5, 6, 7, 8, 9. However, there is no comprehensive evaluation on media service. In view of this, this method is considered to be one-sided, without considering the subjective factors, environmental factors and so on, with the disadvantage of poor portability, which is not suitable for the QoE evaluation on other media services. In this paper, QoE evaluation method based on fuzzy clustering heuristic rules relies on the support of large database to counts and gives an effective evaluation for each evaluation parameter index. When the server carries on QoE evaluation on media service, it will give service score information based on the big data information in media database. Meanwhile, if there is no evaluation information, service score prediction information is given on basis of service transmission status, and inserted into the database as big data source to provide reference to future service evaluation.

2) In end-to-end QoE evaluation method, the client applies active detection technology to periodically send probe packets to detect service evaluation parameters. In multipath transmission QoE evaluation method, Remote Network Monitoring (RMON) is carried out at the client side to report and collect the needed parameter information for service evaluation, which will be sent to the server, in order to ultimately complete the evaluation at the server side. Both the two methods have system overheads, and network transmission overheads caused by media transmission evaluation parameters. However, client system overhead is relatively large in end-to-end QoE evaluation method. QoE evaluation method is required to maintain its own parameters and periodically send QoE evaluation parameters probe packets. In fuzzy clustering heuristic QoE co-evaluation method, the client does not have to care about the cost, which has been transferred to the server to reduce the pressure on the client. As for network transmission overhead, the two methods bear equivalent overhead pressures as a part of the bandwidth is occupied because of parameter information feedback. For this reason, QoE co-evaluation method based on fuzzy clustering heuristic algorithm has certain advantages in system overhead.

3) Service QoE score calculation deviation QD. The purpose of QoE evaluation method is to meet the needs for user experience and give a score, so as to make media transmission control more accurate. In order to give service QoE score deviation, the concept of service QoE score calculation deviation is put forward in this paper. QD is defined as follows:

10 $$QD = \sqrt {\frac{{\mathop \sum \nolimits_{i = 1}^{n} (QoE_{i} - QoE^{'}_{i} )^{2} }}{n}}$$

wherein, QoE_i_ represents the *ith* score value of QoE model, ${\text{QoE}}_{\text{i}}^{'}$ the *ith* actual score of service QoE evaluation, and *n* the number of test period.

**Fig. 12:**
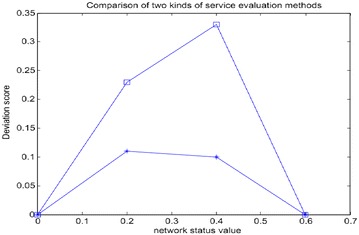
QoE score deviation of different evaluation model conditions

Two conditions of QoE score deviation are given in Fig. 12, which include end-to-end linear model method mentioned in Literature (Chen et al. 2009) and QoE co-evaluation based on fuzzy clustering heuristic method. The network state is excellent when the network value is in the range of (0, 0.2), good when it is in (0.2, 0.4), medium when it is in (0.4, 0.6), and poor when it is in (0.6, 0.8). As can be seen from Fig. 12, the cost of end-to-end calculation deviation of linear model is higher than that of QoE co-evaluation based on fuzzy clustering heuristic method, due to the fact that it cannot completely cover the sample scores.

4) Video codec is an important technical factor for QoE evaluation in the transmission of media service. The existing video streaming codec technology includes H.264, H.265, layered coding, inter layered network coding, and so on (Gogate et al. 2002; Shen et al. 2005; Mao et al. 2003; Lin 2001). Although layered coding and inter layered network coding technologies have received popular recognition in multipath transmission, streaming media do not allow retransmission, considering user experience during transmission. Thus, these media transmission conditions are higher with some challenges.

The existing linear model of service QoE evaluation does not consider the impact of service QoE evaluation mechanism from the perspective of stream codec. In the service QoE evaluation mechanism proposed in this paper, QoE evaluation score mechanism would not be affected when the video streaming is encoded using layered coding and inter layered network coding technologies. Nonetheless, the corresponding QoE evaluation guidance policy requires appropriate guidelines, such as the introduction of partially redundant transmission mechanism, re-distribution of flow payload, and other technologies. It can also select one transport path as a backup path in multipath transmission. When the values of other path network QoS parameters are lower than the critical value, the path shall be prepared for redundant transmission, so as to ultimately improve service QoE score. The research on service QoE policy decision mechanism needs to be further strengthened in the future work.

## Conclusions

In QoE co-evaluation method based on fuzzy clustering heuristic algorithm, the generation rules of service QoE evaluation algorithm are analyzed according to network performance parameters, network flow and parameters distribution in random state are obtained, and then service evaluation strategy algorithm is given according to the analysis results. The design of heuristic evaluation algorithm is based on two kinds of evaluation rules, to deal with media parameters under different conditions, so as to get service evaluation rules of the process. With system clustering method, fuzzy clustering analysis of media database is conducted to analyze media parameters, and finally acquire service scoring information. With the server as the core, QoE co-evaluation method based on fuzzy clustering algorithm adopts centralized management modes, considering media service QoE evaluation from many aspects to effectively avoid the problems caused by the fact that only the client side maintains the database in end-to-end QoE evaluation, such as lack of data and delayed database update. Hence, this method improves media transport QoE evaluation accuracy and efficiency. In the simulation, the proposed method and the original end-to-end linear evaluation method are compared and analyzed, and the experiments have verified the effectiveness and feasibility of this scheme.

## References

[CR1] Bhaskara T, Pal MN, Palb AK (2011). A heuristic for RCPSP with fuzzy actitity times [J]. Eur J Oper Res.

[CR2] Brooks P, Hestnes B (2010). User measures of quality of experience: why being objective and quantitative is important [C]. IEEE Netw.

[CR3] Chen KT, Tu CC, Xiao WC (2009). One click: a framework for measuring network quality of experience [C]. IEEE INFOCOM.

[CR4] Garcia M, Canovas A, Edo M et al (2009) A QoE management system for ubiquitous IPTV devices. In: Third international conference on mobile ubiquitous computing, systems, services and technologies, UBICOMM ‘09. IEEE, Sliema, vol 26, no 3, pp 147–152

[CR5] Gogate N, Panwar SS (1994) On a resequencing model for high speed networks. In: IEEE proceedings on networking for global communications, INFOCOM ‘94. IEEE, Toronto, vol 1, pp 40–47

[CR6] Gogate N, Panwar SS (1999). Assigning customers to two parallel servers with resequencing [J]. IEEE Commun Lett.

[CR7] Gogate N, Chung DM, Panwar ss (2002). Supporting image and video applications in a multihop radio environment using path diversity and multiple description coding [J]. IEEE Trans Circuits Syst Video Technol.

[CR8] 3GPP (2004) TR 23.917. Dynamic policy control enhancements for end-to-end QoS V1.2.0 [EB/OL]. http://www.3gpp.org/DynaReport/23917.htm. Accessed 22 Jan 2004

[CR9] 3GPP (2007) TS 29.208. End-to-end quality of service (QoS) signaling flows V6.7.0 [EB/OL]. http://www.3gpp.org/DynaReport/29208.htm. Accessed 22 June 2007

[CR10] Hall M, Frank E, Holmes G (2009). The WEKA data mining software: an update [J]. ACM SIGKDD Explor Newsl.

[CR11] IETF (2011) Threat analysis for TCP extensions for multipath operation with multiple addresses [EB/OL]. http://pike.lysator.liu.se/docs/ietf/rfc/61/rfc6181.xml. Accessed 20 Mar 2011

[CR12] ITU-T (2007) Definition of quality of experience (QoE) [EB/OL]. http://www.ties.itu.ch/ftp/public/itu-t/fgiptv/readonly/Previous_Meetings/20070122_MountainView/il/T05-FG.IPTV-IL-0050-E.htm. Accessed 22 Jan 2007

[CR13] Karagiannis T, Molle M, Faloutsos M, Broido A (2004). A nonstationary Poisson view of Internet traffic [C]. Proc 23rd Annu Jt Conf IEEE Comput Commun Soc.

[CR14] Kim HL, Choi SG (2010). A study on a QoS/QoE correlation model for QoE evaluation on IPTV service [C]. 12th Int Conf Adv Commun Technol (ICACT).

[CR15] Kumar U, Oyman O (2013). QoE evaluation for video streaming over eMBMS [J]. J Commun.

[CR16] Lei W, Zhang W, Liu S (2014a) A framework of multipath transport system based on application-level relay (MPTS-AR) draft-leiwm-tsvwg-mpts-ar-02 [EB/OL]. http://www.datatracker.ietf.org/doc/draft-leiwm-tsvwg-mpts-ar/. Accessed 24 Jan 2015

[CR17] Lei W, Zhang W, Liu S (2014b) Multipath real-time transport protocol based on application-level relay (MPRTP-AR) draft-leiwm-avtcore-mprtp-ar-02[EB/OL]. http://www.datatracker.ietf.org/doc/draft-leiwm-avtcore-mprtp-ar/. Accessed 24 Jan 2015

[CR18] Lei W, Liu S, Zhang W (2014c) Multipath message transport protocol based on application-level relay (MPMTP-AR) draft-leiwm-tsvwg-mpmtp-ar-02[EB/OL]. http://www.datatracker.ietf.org/doc/draft-leiwm-tsvwg-mpmtp-ar/. Accessed 26 Jan 2015

[CR19] Leland WE, Taqqu MS, Willinger W, Wilson DV (1994). On the self-similar nature of ethernet traffic (extended version) [J]. IEEE/ACM Trans Netw.

[CR20] Lin S, Mao S, Wang Y (2001). A reference picture selection scheme for video transmission over Ad-Hoc networks using multiple paths [J]. IEEE Int Conf Multimed Expo.

[CR21] Lu L, Liang Y, Yang H, et al (2012) Danger theory: a new approach in big data analysis [C]. In: International conference on automatic control and artificial intelligence (ACAI 2012). IET, Xiamen, pp 739–742

[CR22] Mao S, Lin S, Panwar SS (2003). Video transport over ad hoc networks: multistream coding with multipath transport [J]. IEEE J Sel Areas Commun.

[CR23] Mao S, Lin S, Wang Y (2005). Multipath video transport over ad hoc networks [J]. IEEE Wirel Commun.

[CR24] Mao S, Panwar SS, Hou YT (2005). On optimal partitioning of realtime traffic over multiple paths [C]. INFOCOM 2005 24th Annu Jt Conf IEEE Comput Commun Soc.

[CR25] Msakni HG, Yousef H (2012) Provisioning QoE over converged networks: issues and challenges [C]. In: 2012 IEEE 14th international conference on high performance computing and communication (HPCC) and 2012 IEEE 9th international conference on embedded software and systems (ICESS). IEEE, Liverpool, pp 891–896

[CR26] Ning Z, Guo L, Wang X (2012). Joint scheduling and routing algorithm with load balancing in wireless mesh networks [J]. Comput Electr Eng.

[CR27] Paxson V, Floyd S (1995). Wide-area traffic: the failure of Poisson modeling [J]. IEEE/ACM Trans Netw.

[CR28] Shen Y, Liu Z, Panwar SS (2005). Streaming layered encoded video using peers [C]. IEEE Int Conf Multimed Expo.

[CR29] Song Q, Ning Z, Wang S (2012). Link stability estimation based on link connectivity changes in mobile Ad hoc networks [J]. J Netw Comput Appl.

[CR30] Sterle J, Volk M, Sedlar U (2011). Application-based NGN QoE controller [J]. IEEE Commun Mag.

[CR31] Sterle J, Volk M, Sedlar U (2011). Application-based NGN QoE controller [J]. IEEE Commun Mag.

[CR32] Volk M, Sterle J, Sedlar U (2010). An approach to modeling and control of QoE in next generation networks [J]. IEEE Commun Mag.

[CR33] Wang S, Wang HJ, Qin XP (2011). Architecting Big data: challenges, studies and forecasts [J]. Chin J Comput.

[CR34] Yu P, Zeng H, Rui L (2011). A novel QoE assessment method for wireless networks [C]. Adv Intell Aware Internet (AIAI 2011).

[CR35] Zhang J, Ansari N (2011). On assuring end-to-end QoE in next generation networks: challenges and a possible solution [J]. IEEE Commun Mag.

[CR36] Zhou L, Yang Z, Wen Y (2013). Resource allocation with incomplete information for QoE-driven multimedia communications [J]. IEEE Transact Wirel Commun.

